# Circulating hsa-miR-320a and its regulatory network in type 1 diabetes mellitus

**DOI:** 10.3389/fimmu.2024.1376416

**Published:** 2024-10-11

**Authors:** Rasheeba Nizam, Md Zubbair Malik, Sindhu Jacob, Osama Alsmadi, Heikki A. Koistinen, Jaakko Tuomilehto, Hessa Alkandari, Fahd Al-Mulla, Thangavel Alphonse Thanaraj

**Affiliations:** ^1^ Department of Genetics and Bioinformatics, Dasman Diabetes Institute, Dasman, Kuwait; ^2^ Department of Medicine, University of Helsinki and Helsinki University Hospital, Helsinki, Finland; ^3^ Department of Public Health and Welfare, Finnish Institute for Health and Welfare, Helsinki, Finland; ^4^ Metabolism Group, Minerva Foundation Institute for Medical Research, Helsinki, Finland; ^5^ Department of Public Health, University of Helsinki, Helsinki, Finland; ^6^ Diabetes Research Group, King Abdulaziz University, Jeddah, Saudi Arabia; ^7^ Department of Population Health, Dasman Diabetes Institute, Kuwait City, Kuwait; ^8^ Department of Pediatrics, Farwaniya Hospital, Ministry of Health, Kuwait City, Kuwait

**Keywords:** hsa-miR-320a-3p, miRNA, type 1 diabetes, Kuwait, genetics, next-generation sequencing, weighted gene co-expression network analysis

## Abstract

**Introduction:**

Increasing evidence from human and animal model studies indicates the significant role of microRNAs (miRNAs) in pancreatic beta cell function, insulin signaling, immune responses, and pathogenesis of type 1 diabetes (T1D).

**Methods:**

We aimed, using next-generation sequencing, to screen miRNAs from peripheral blood mononuclear cells of eight independent Kuwaiti-Arab families with T1D affected siblings, consisting of 18 T1D patients and 18 unaffected members, characterized by no parent-to-child inheritance pattern.

**Results:**

Our analysis revealed 20 miRNAs that are differentially expressed in T1D patients compared with healthy controls. Module-based weighted gene co-expression network analysis prioritized key consensus miRNAs in T1D pathogenesis. These included hsa-miR-320a-3p, hsa-miR-139-3p, hsa-miR-200-3p, hsa-miR-99b-5p and hsa-miR-6808-3p. Functional enrichment analysis of differentially expressed miRNAs indicated that PI3K-AKT is one of the key pathways perturbed in T1D. Gene ontology analysis of hub miRNAs also implicated PI3K-AKT, along with mTOR, MAPK, and interleukin signaling pathways, in T1D. Using quantitative RT-PCR, we validated one of the key predicted miRNA-target gene-transcription factor networks in an extended cohort of children with new-onset T1D positive for islet autoantibodies. Our analysis revealed that hsa-miR-320a-3p and its key targets, including *PTEN, AKT1, BCL2, FOXO1* and *MYC*, are dysregulated in T1D, along with their interacting partners namely *BLIMP3, GSK3B*, *CAV1, CXCL3, TGFB*, and *IL10*. Receiver Operating Characteristic analysis highlighted the diagnostic potential of hsa-miR-320a-3p, *CAV1, GSK3B* and *MYC* for T1D.

**Discussion:**

Our study presents a novel link between hsa-miR-320a-3p and T1D, and highlights its key regulatory role in the network of mRNA markers and transcription factors involved in T1D pathogenesis.

## 1 Introduction

Type 1 diabetes (T1D) is an autoimmune disease characterized by an unfavorable immune response against pancreatic beta cells, which leads to insulin deficiency and overt hyperglycemia. The etiology of T1D remains unclear, yet several genetic, immunological, and environmental factors are associated with the disease. A genetic basis for T1D has been evidenced by 78 genome-wide regions associated with the disease (1–4). Human leukocyte antigen (HLA) is by far the strongest predictor and accounts for at least 50% of the heritability in T1D (5). The familial aggregation of T1D, especially clustering among first-degree relatives, indicates strong genetic basis for the disease (6, 7). However, only a negligible percentage of T1D cases represent monogenic forms characterized by either a dominant, recessive, or X-linked pathogenic variant (8–10). The occurrence of T1D phenotypic discordance in monozygotic twins and the incidence of T1D sporadic cases with no parent-to-child inheritance pattern suggest a greater role for gene–environment interactions in triggering the disease (7). Accordingly, several environmental and lifestyle factors, such as viral infections, toxicity exposure, microbial dysbiosis and dietary choices during infancy, have been associated with T1D onset (11), but these have not been unequivocally proven to be causal.

Recent years have witnessed a growing interest in studies utilizing microRNA (miRNA) as biomarkers for the early prediction of T1D. Dysregulation of miRNA is associated with pancreatic beta cell function, insulin signaling, and immune response (12). Studies using peripheral blood mononuclear cells (PBMC) from patients with T1D have observed dysregulation of key miRNAs, such as miR-21, miR-93 and miR-326, and thereby indicated their potential impact on inflammatory and autoimmune responses (13). In T1D animal models, overexpression of miR-21 interferes with the β-cell development (14). Upregulation of miR-29 in both animal and human pancreatic islets has been observed to disrupt the beta cell function and glucose-induced insulin secretion (15–18). Similarly, miRNAs have also been implicated in cytokine-mediated beta cell destruction, as evidenced by the deregulated expression of miR-21-5p, miR-30b-3p, miR-34, miR-101a and miR-146a-5p in response to inflammatory cytokines such as IL-1β and TNF in MIN6 cells and human pancreatic islets (19, 20). These studies collectively indicate that miRNAs play a potential role in T1D pathogenesis and warrant further in-depth studies on the dysregulation of miRNAs in T1D pathogenesis.

To gain further knowledge on the role of miRNA in T1D pathogenesis, we aimed to identify the key miRNAs involved in T1D by utilizing next-generation sequencing technologies in a familial cohort consisting of siblings with T1D characterized by no parent-to-child inheritance pattern. As miRNA expression is confounded by several factors such as diet, environment, lifestyle, and ethnicity (21, 22), we considered that adopting a sib-pair study design may, by way of minimizing the impact of confounders and enriching for disease parameters, lead to the identification of unique genetic markers associated with T1D. We also aimed to validate the shortlisted miRNA markers in a unique set of sporadic T1D cases, with no vertical or horizontal transmission of the disease, to ensure generalizability of the results. We further aimed to identify, by way of performing module-based weighted gene co-expression network analysis (WGCNA), the key regulatory network consisting of miRNA markers, mRNA markers, and transcription factors (TFs) involved in T1D pathogenesis.

## 2 Methods

**Figure 1:**
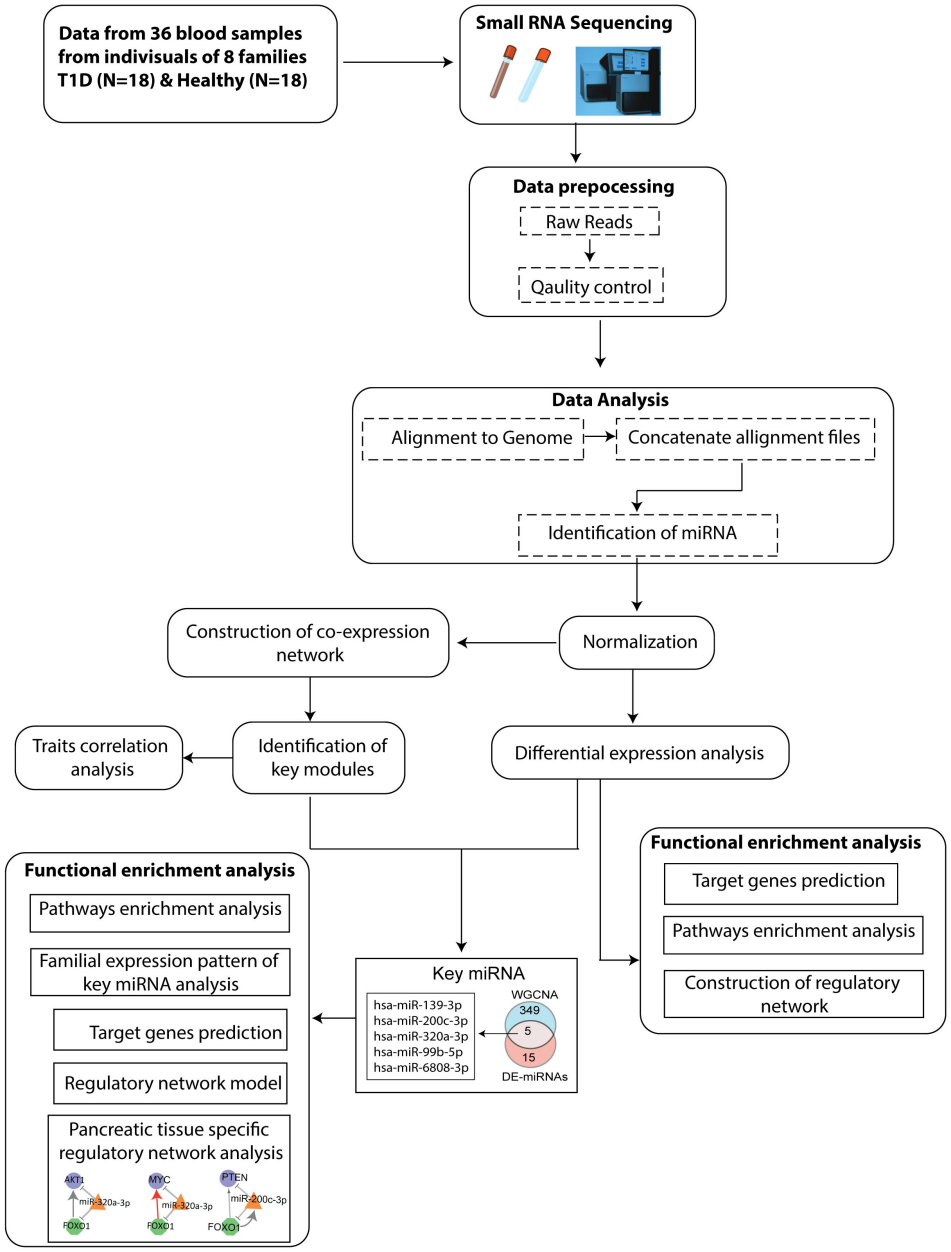
Flowchart depicting the steps of miRNA sequencing data used to identify key miRNAs, followed by downstream functional enrichment analysis.

The Schematic workflow of this study is shown in **Figure 1**.

### 2.1 Study design and clinical recruitment

This study was approved by the ethical committee of Dasman Diabetes Institute and was performed in accordance with the principles of the Declaration of Helsinki, as revised in 2008. Written informed consent was obtained from all study participants. In cases of children, informed consent was obtained from the parents/legal guardians, and assent was obtained from children aged seven years and more.

Samples used in this study were obtained from the Childhood-Onset Diabetes eRegistry (CODeR) (23) maintained by Dasman Diabetes Institute in collaboration with the Ministry of Health (MOH) of Kuwait. A total of eight families consisting of 18 people with T1D and 18 unaffected first-degree relatives were recruited for the present study (**Table 1**). Selection criteria included the following: (i) families with a minimum of two T1D cases exhibiting a horizontal transmission of the disease, (ii) diagnosis of T1D confirmed using World Health Organization criteria, which include fasting hyperglycemia and absolute insulin deficiency, as defined by low C-peptide concentration (<0.3 nmol/l), (iii) T1D characterized by presence of one or more autoantibodies against pancreatic islet cells, and (iv) T1D people of Kuwaiti-Arab origin.

**Table 1:** Clinical characteristics of individuals with T1D from eight recruited families.

Family No	No. of Affected/Unaffected members	Relationship (status)	Age	Age at onset of T1D	T1D duration	Sex	BMI (kg/m^2^)	BP	HbA1C (%)	Plasma glucose (mmol/l)	T. Chol. (mmol/l)	LDL (mmol/l)	HDL (mmol/l)	TGL (mmol/l)	Uric acid µmol/L	U. MA/Cr. (mg/g creat.)
								Systolic/diastolic								
								(mmHg)								
F1	3/3	Daughter (Affected)	12.6	6	6.6	F	26.6	126/69	11		5.3	3	1.52	1.62	198	11.1
		Daughter (Affected)	12.6	6	6.6	F	20.9	122/71	10.2						172	6.9
		Son (Affected)	7	5	2	M	17	124/63	10.1		4.8	3	1.58	0.47	121	
F2	2/3	Son (Affected)	13.6	2	11.6	M	25	124/76	8.4	24.4	4.6	2.5	1.31	1.76	200	5.9
		Daughter (Affected)	20	12	10	F	18.8	115/70	7	3.7	4.7	3	1.29	0.87	124	9.8
F3	2/1	Daughter (Affected)	10.1	3	7.1	F	18.4	103/58	8.2	11.1	4.4	2.5	1.66	0.47	158	47.5
		Son (Affected)	21.2	3	18.2	M	24.2	129/67	7.3	12.7	4	2.6	1.01	0.76	294	2.7
F4	2/2	Son (Affected)	8	3	5	M	18.9	109/50	9.3	13	4.2	2.5	1.48	0.5	112	
		Son (Affected)	12.4	9	3.4	M	24.4	126/71	8.6		4.2	2.7	1.28	0.49		
F5	2/3	Son (Affected)	14.7	6	8.7	M	26.7	128/60	10.3	15.6	5	2.9	1.43	1.4	199	4.2
		Son (Affected)	4.7	4	0.7	M	14.4	97/66	8.7	19.5	4	2.1	1.33	1.28	148	
F6	3/1	Daughter (Affected)	8.7	1.4	7.3	F	13.5	123/57	9.7	17.8	5.7	3.1	2.35	0.49	206	8.1
		Son (Affected)	11.7	6	5.7	M	19.2	114/58	10.3	13.9	4.6	3	1.35	0.47	188	5.6
		Daughter (Affected)	13.5	5	8.5	F	22	122/69	11.3	17.2	5	3.4	1.24	0.9	243	5.1
F7	2/2	Daughter (Affected)	17.4	1.8	15.6	F	23.2	113/63	8.2	14.2	3.5	2.1	1.26	0.3	156	2.9
		Daughter (Affected)	15.2	2.2	13	F	22.9	110/65	11.4	15	5.1	2.5	2.08	1.06	176	10.6
F8	2/3	Daughter (Affected)	8.2	8	0.2	F	18.2	120/71								
		Daughter (Affected)	5	4	1	F	15.1	120/66	11.2	4.1	5.7	3.2	2.32	0.34	98	

The validation cohort consisted of 110 T1D sporadic cases and 15 controls without T1D. Selection criteria included: (i) sporadic cases with no parent-to-child or horizontal transmission of the disease (ii) diagnosis of T1D confirmed based on World Health Organization criteria, (iii) T1D characterized by the presence of autoantibodies against pancreatic islet cells, and (iv) people of Kuwaiti-Arab origin. This cohort included 60 male, and 50 female sporadic T1D cases with an average age of 12 ± 3.5 years, body mass index (BMI) of 20.6 ± 4.9 kg/m^2^, glycated hemoglobin A1c (HbA1c) of 9.4 ± 1.71%, and plasma glucose of 12.1 ± 5.49 mmol/l at baseline. The healthy control subjects were ethnically matched Kuwaiti-Arab individuals (n=15) with no prior medical history of any chronic debilitating disease. This included 10 male and 5 female volunteers with an average age of 27± 5.3 years, BMI of 30.2 ± 6.4 kg/m^2^ and plasma glucose of 5.4 ± 0.54 mmol/l. Of the samples from this validation cohort, miRNA samples were available in sufficient quantities in a set of 52 sporadic T1D children and 10 ethnically matched controls. While the miRNA markers were validated in this subset of validation cohort, the mRNA markers were validated in the entire validation cohort. Blood samples were collected at the clinics of Dasman Diabetes Institute. The date of the first insulin injection was taken as the date of the onset of T1D. The collected data included age, sex, BMI, nationality, date of birth, date of T1D diagnosis, family history of diabetes in first-degree relatives, and measurements of HbA1c, plasma glucose, blood pressure, serum uric acid, blood urea nitrogen, and creatinine concentrations.

### 2.2 miRNA isolation, libarary preparation, sequencing and identification of differentially expressed miRNAs

The extraction of miRNA from PBMCs was performed using the miRNeasy kit (Qiagen, Hilden, Germany) according to the manufacturer’s protocol. Quantification of miRNA was carried out using the miRNA assay kit on a qubit fluorometer (Thermofisher Scientific, Massachusetts, United States).

A total of 10ng of purified miRNA samples was used for library preparation. miRNome-wide sequencing libraries were prepared using the QIAseq miRNA Library Kit (Qiagen, Hilden, Germany) according to the manufacturer’s instructions (24, 25). The protocol involves sequential ligation of 3′ and 5′ end adapters followed by universal cDNA synthesis with unique molecular index assignment, cDNA cleanup, library amplification and library cleanup using QMN beads. The prepared libraries were validated and quantified using bioanalyzer (Agilent, California, United States) and qubit fluorometer (Thermofisher Scientific, Massachusetts, United States), respectively. Sequencing was carried out on MiSeq system using MiSeq 150-cycle version 3 kit (Illumina Inc. USA).

GeneGlobe data analysis tool is a supportive RNA-seq data analysis solution powered by Qiagen, included with the small RNA seq library kits. The portal initially removes low quality bases and reads without 3’ adapters using cutadapt (cutadapt.readthedocs.io/en/stable/guide.html); reads with less than 16 bp insert sequences or less than 10 bp unique molecular indices (UMI) sequences are excluded from the analysis. The obtained reads are mapped against miRBase V21 (https://mirbase.org/) where up to two mismatches are tolerated using bowtie (bowtie-bio.sourceforge.net/index.shtml). Normalization is carried out based on UMI with a *p*-value threshold of <0.05 and |log fold change (FC)| ≥ or < 1.0. The resulting Fastq files were used for differential miRNA expression analysis using the GeneGlobe data analysis tool. We performed both family-based distinct and concatenate analysis to identify DE miRNAs in T1D individuals compared with unaffected family members using the GeneGlobe data analysis tool based on unique molecular indices with a *p*-value threshold of <0.05 and |log fold change (FC)| ≥ or < 1.0. The resulting data were visualized by generating volcano plots and heatmaps using ggplot2 and pheatmap packages, respectively.

### 2.3 Functional enrichment analysis of key miRNAs

Enrichment analysis of DE miRNA data to identify the target regulatory genes was carried out using MIENTURNET (26–28), which is a web tool that predicts miRNA-target interactions by performing statistical analysis on computationally predicted, and experimentally validated data from miRTarBase (29), miRDB (30) and TargetScan (31) databases. Significantly correlated pairs of interacting DE miRNAs and mRNAs were included to create co-expression networks using Cytoscape 3.6.1 (32). Pathway analysis of DE miRNAs was performed using the MIENTURNET tools (26). A *p*-value < 0.05 was used as a cut-off for false discovery rate (FDR) to detect significantly enriched pathways. The statistically most enriched Gene Ontology (GO) terms were visualized in ggplot2 (33).

### 2.4 Co-expression network analysis and module detection

The WGCNA R (34) software package was used to perform weighted gene co-expression network analysis on DE miRNA data to construct a co-expression network, identify the key modules, relate them to clinical data, and delienate the key biomarkers involved in the pathogenesis of T1D. Prior to performing network construction and module detection, samples were clustered and visualized in a heatmap to examine how clinical traits relate to the sample dendrogram (**Supplementary Figure S1**). In co-expression analysis, biologically meaningful gene pairs are characterized by high correlations (signal) compared to random gene pairings that are usually characterized by low correlation (noise). Firstly, the miRNA expression similarity matrix was constructed by calculating the absolute value of Pearson’s correlation coefficient between miRNA pairs. This similarity matrix was then converted into an adjacency matrix using a power adjacency function, which encodes the strength of the connection between node pairs. According to the scale-free topological algorithm, the adjacency matrix met the scale-free topology criterion when the R^2^ value approximated 0.80 (**Supplementaryry Figures S1B, C**). The adjacency matrix was subsequently converted into a topological matrix, using the topological overlap measure (TOM) to describe the degree of association between miRNAs. TOM indicates the degree of dissimilarity between miRNA pairs. Hierarchical clustering was performed using 1-TOM as a distance measure, and modules of co-expressed miRNAs were identified using the dynamic tree cut procedure with a minimum size cutoff of 5. Highly similar modules were then merged using the Merge Dynamic function.

The Eigengene network tool was utilized to investigate module associations with biological data. We used module eigengene (ME), the first principal component of module expression, to represent the expression profile of module miRNAs. Relevance of each miRNA is assessed by computing the following parameters: the gene significance (GS), the module membership (MM), and Module Connectivity (MC). MC is typically calculated by averaging the gene significance (GS) of all the genes within the module. Gene significance reflects the correlation between the expression of a gene and the trait of interest. A value of 0 for gene significance indicates that the gene is not significant with regard to the biological question of interest. GS can take on positive or negative values. Module significance measures how strongly the genes within a particular module are associated with a specific trait or phenotype. A higher value for module significance suggests that the module, as a whole, is more strongly associated with the phenotype, making it biologically relevant. Module Membership (MM, also known as KME or Eigengene-based Connectivity) quantifies how well each individual gene correlates with the eigengene of its module. An eigengene is the first principal component of the module’s gene expression data and serves as a representative profile of the module. MM is calculated as the correlation between the expression profile of a gene and the module eigengene. Genes with high MM values (close to 1 or -1) are considered highly connected or as core genes within the module and are likely to play a central role in the module’s biological functions. In this study, potential key miRNAs were identified as those within a given module that were highly connected (having the highest absolute MM) and showed the strongest correlation with the trait of interest (having the highest absolute GS). A threshold of 0.7 was applied for both MM and GS. Module connectivity refers to the degree of connection a gene has with other genes within the same module, indicating how central a gene is within the module’s co-expression network. Connectivity is computed as sum of adjacencies (co-expression similarity) between a gene and all other genes in the module. Genes with high intramodular connectivity are considered hub genes. These hub genes are important because they may regulate key biological processes within the module.

Therefore to validate module-trait relationships (MTRs), defined as the correlation between MEs and clinical features of miRNA modules, we categorized miRNAs into matching modules according to the constructed modules (34, 35). We calculated the ME of each module and included the related clinical features. We further calculated miRNA significance defined as the log_10_-transformation of *p*-value in the linear regression slope between gene expression and clinical features), and module significance (MS) (described as the average miRNA significance of all miRNAs in the module) to further assess correlation intensity between a miRNA module and clinical features such as age, BMI, HbA1C, plasma glucose, alanine aminotransferase (ALT), aspartate aminotransferase (AST), serum total cholesterol, low-density lipoprotein (LDL) cholesterol, high-density lipoprotein (HDL) cholesterol, and calcium.

### 2.5 Feedforward loops of miRNA-transcription factor-gene network

We further constructed miRNA‐Transcritpion Factor (TF) feedback loops and miRNA‐TF‐gene Feedforward loops (FFLs) using FFL tool webserver (36) and visualized their regulatory networks using Cytoscape 3.6.1 (32). The miRNA-long noncoding RNA (lncRNA) interaction analysis was carried out with DIANA-LncBaseV3.0 tool using Ensembl and Refseq databases (37).

### 2.6 Validation of key targets by quantitative real-time PCR

Regulatory target genes of miRNA were validated using specific TaqMan gene expression assays (Thermofisher Scientific, Massachusetts, United States) on a quantitative real-time PCR system (Quant Studio6, Thermofisher Scientific, Massachusetts, United States). RNA was extracted from peripheral blood using Qiagen RNA blood mini kit (Qiagen, Hilden, Germany), reverse transcribed using ABI reverse transcriptase kit (Applied Biosystem, USA) and quantitative real-time PCR was performed using pre-designed ready-to-use miRCURY LNA miRNA PCR assay (hsa-miR-320a-3p, #YP00206042) relative to 5S rRNA (#YP00203906). Target gene validation was performed using TaqMan gene expression assays *PTEN* (Hs02621230_s1), *AKT1* (Hs00178289_m1), *BCL2* (Hs04986394_s1), *FOXO1* (Hs00231106_m1), *MYC* (Hs00153408_m1), *BLIMP3* (Hs00153357_m1), *GSK3B* (Hs00275656_m1), *CAV1* (Hs00971716_m1), *CXCL3* (Hs00171061_m1), *IL-10* (Hs00961622_m1), *TGFB* (Hs00998133_m1) and relative to *GAPDH* (Hs02786624_g1) as endogenous control on ABI 7500 real-time PCR system following manufacturer’s protocol.

### 2.7 Statistical analysis

The fold change (FC) was calculated using the 2 − ΔΔCT method, and differences in the expression levels between the two tested groups were detected using Mann-Whitney U-test. Correlation between variables were calculated using Spearman’s rank correlation test and were considered statistically significant at *p*-value <0.05. Receiver Operating Characteristic (ROC) analysis was based on a logistic regression (38) considering the shortlisted hsa-miR-320a-3p and its interactive mRNA partners, such as *CAV1, GSK3B* and *MYC*, as potential predictors. To determine the ideal biomarker combinations, both a single marker and a combinatorial analysis were used. A cross-validation (CV) procedure was employed to provide an unbiased estimate of biomarker performance (39). Multiple rounds of CV were conducted resulting in a series of ROC curves, to ensure a reliable performance estimate by using R.4.4.1. The performance results were averaged over these rounds and a 10-fold CV strategy was adopted to compare different models.

## 3 Results

A total of eight Kuwaiti-Arab T1D families, who showed no parent-to-child transmission of the disease, were initially examined in this study. This included 18 people with T1D, 10 of whom were female and eight were male. The clinical characteristics of 18 people with T1D are shown in **Table 1**. The average age at the time of recruitment and age at onset of T1D cases were 12 ± 4.7 and 4.7 ± 3.0 years, respectively. The in-family control set included 18 individuals (10 females and 8 males) with an average age of 31 ± 16.6 years and were with no prior medical history of chronic debilitating diseases. The average duration of T1D among our patients was 7.1 ± 5.1 years. The average body mass index of T1D case and control subjects were 20.5 ± 4.1 kg/m^2^ and 26.4 ± 7.9 kg/m^2^, respectively.

None of our patients showed elevated levels of lipids though minor variations were observed within the borderline range. Only a 10-year-old female patient from family 3 was observed to be positive for anti-endomysial Ab (AEA), anti-TPO antibodies (363.4 IU/ml), anti-Tissue Transglutaminase IgG (15.2 IU/ml), and anti-Tissue Transglutaminase (IgA >200 IU/ml) tests indicating the presence of Hashimoto thyroiditis and Celiac Disease. The patient also showed a high urine albumin-creatinine ratio (47.5 mg/g) indicative of an early-stage kidney disease. None of the other tested patients was positive for anti-endomysium antibody, anti-thyroid peroxidase antibody and anti-tissue transglutaminase tests. Similarly, hyperuricemia was also not reported in any of the tested patients.

**Figure 2:**
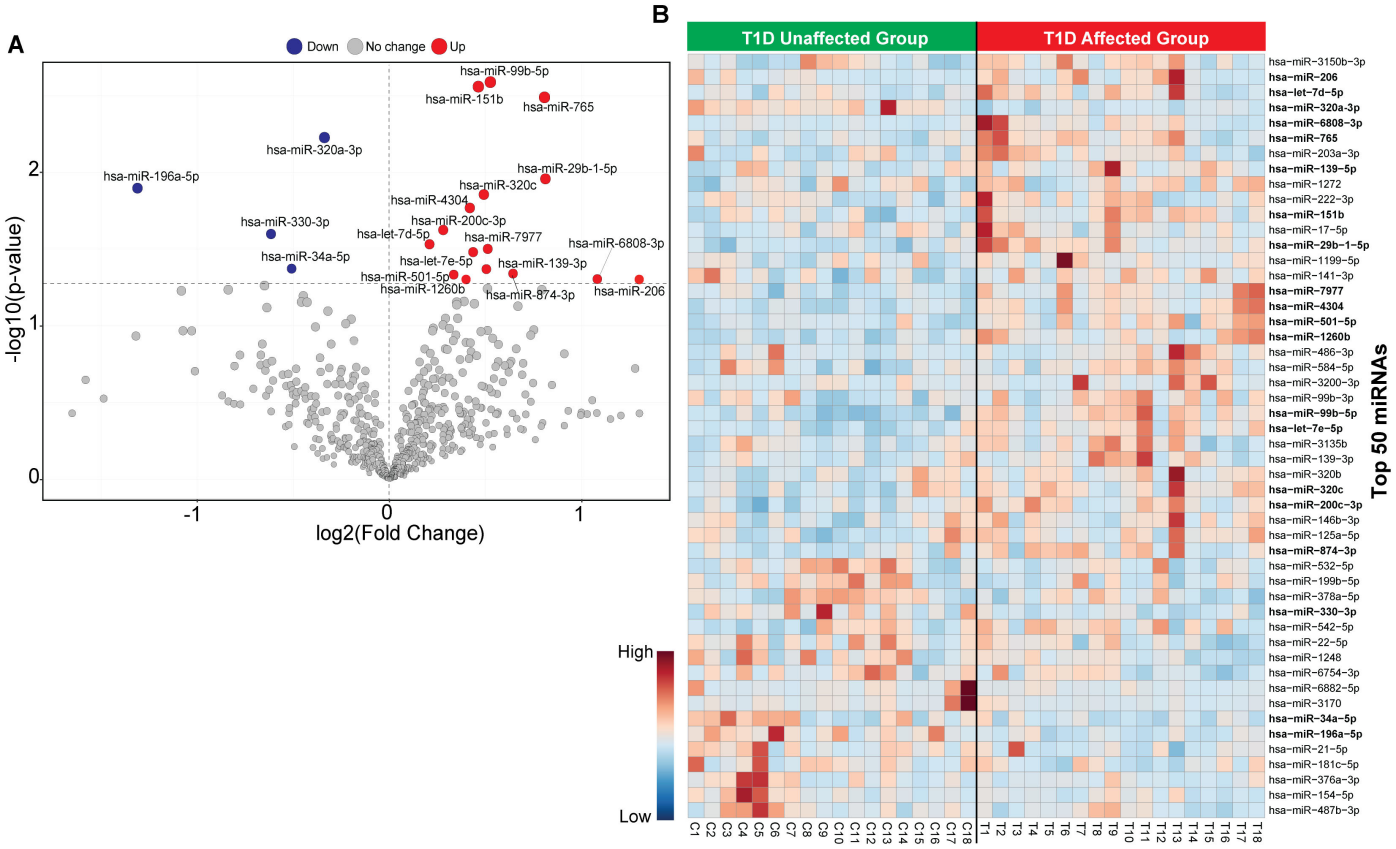
Classification of miRNA based in T1D affected versus non-diabetic individuals. **(A)** The volcano plot presenting differential expression of miRNA with a threshold of false discovery rate (FDR)* p-value<* 0.05 and |log2 fold change (FC)| ≥ or < 1.0. The red dot represents upregulated, the blue dot represents downregulated and the grey dot represents unaffected miRNA targets. **(B)** Heatmap of the top 50 circulating miRNAs across all samples for unaffected T1D and affected T1D groups. Red color shows the upregulated and blue color shows the downregulated miRNAs.

**Figure 3:**
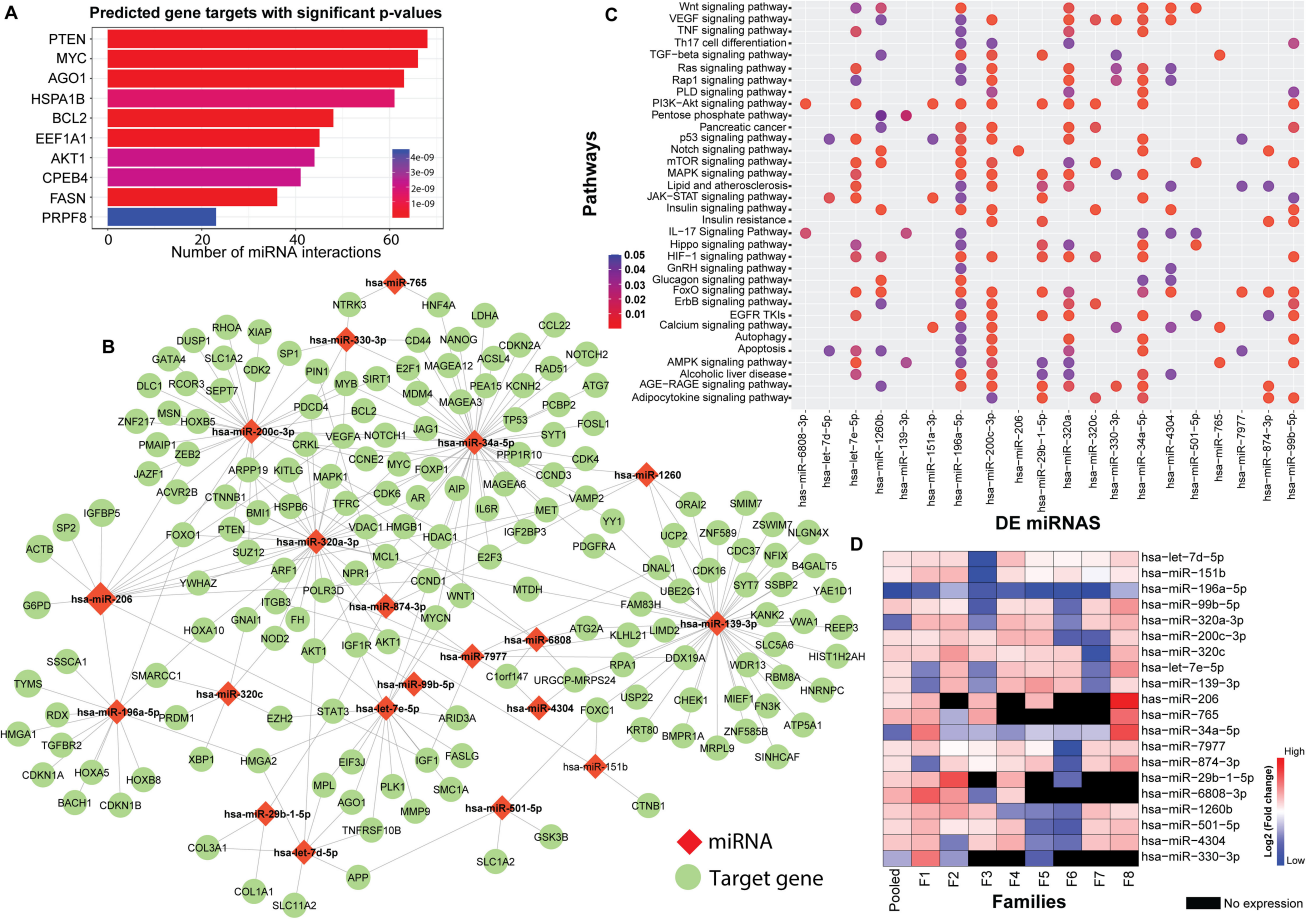
Genetic Perturbations Revealed by Regulatory Networks analysis. **(A)** miRNA–mRNA target interaction network obtained from the DE miRNAs shortlisted from the eight tested families with T1D. The bar plot represents each target gene resulting from the enrichment analysis along with the count of interacting miRNAs. Colors of the bars represent the adjusted *p*-values (FDR). **(B)** Significant miRNA–mRNA target interaction network retrieved from MIENTURNET based on DE miRNAs form the eight tested families with T1D. The miRNAs are represented by red diamond shaped nodes and the target genes are represented by blue dots. **(C)** Dot plots resulting from functional enrichment analysis indicating the pathways that are significantly dysregulated. The y-axis reveals the annotation classifications, the x-axis presents the miRNAs, and the plotted data points as circles represent the count of identified targets. Sizes of the circles correspond to the count of DE miRNAs whereas the colors of the circles indicate the adjusted *p*-values. **(D)** The heatmap shows family-based analysis wherein the log fold changes in the expression of the 20 DE miRNAs in individual T1D families, and a pooled analysis is depicted. F1-F8 represents each individual family. Red colors represent upregulated, blue colors show downregulated miRNAs and black colors represent miRNAs that are not expressed.

The flowchart indicating the steps from miRNA sequencing to identification of key miRNAs followed by downstream functional enrichment analysis is presented in **Figure 1**. An average of 1,961,698 sequencing reads were obtained per sequenced sample and 615 unique miRNAs were detected in each of the tested case and control groups (**Supplementary Table S1**). Volcano plots representing significant DE miRNAs between T1D affected and unaffected members are shown in **Figure 2A** (**Supplementary Table S2**). We visualized the top 50 DE miRNAs using heatmaps (**Figure 2B**). Significant differences were observed in the expression levels of miRNA between people with T1D and non-diabetic controls. We identified 20 unique miRNAs that are significantly DE between members of T1D affected versus unaffected groups with a *p*-value cut-off of <0.05, and |log fold change (FC)| ≥ or < 1.0. (**Figure 2B**). The miRNA-gene expression analysis of the key DE miRNAs predicted 10 hub genes, namely *PTEN, MYC, AGO1, HASPA1B, BCL2, EEEF1A1, AKT1, CPEB4, FASN*, and *PRPF8*, that are significantly deregulated in T1D individuals (FDR *p*-value ≤0.05) (**Figure 3A**). *PTEN* and *MYC* are the top two genes impacted by the DE miRNAs. The key miRNA–mRNA target interaction network retrieved from MIENTURNET are presented in **Figure 3B** (**Supplementary Table S3**). Functional enrichment analysis revealed 41 significant pathways that were differentially regulated by the shortlisted miRNAs (FDR *p*-value ≤0.05) (**Figure 3C**; **Supplementary Table S4**). PI3K-Akt signaling was the top enriched pathway shortlisted by our NGS-based pathway analysis. In addition, we depicted the differences in the expression profiles of key shortlisted miRNAs in tested individual families. The heatmap presents fold change calculated for every case-control pair from each distinct families tested, indicating log fold changes of 20 miRNAs that were differentially expressed (**Figure 3D**).

### 3.1 Module-based computational analysis of miRNA in T1D

**Figure 4:**
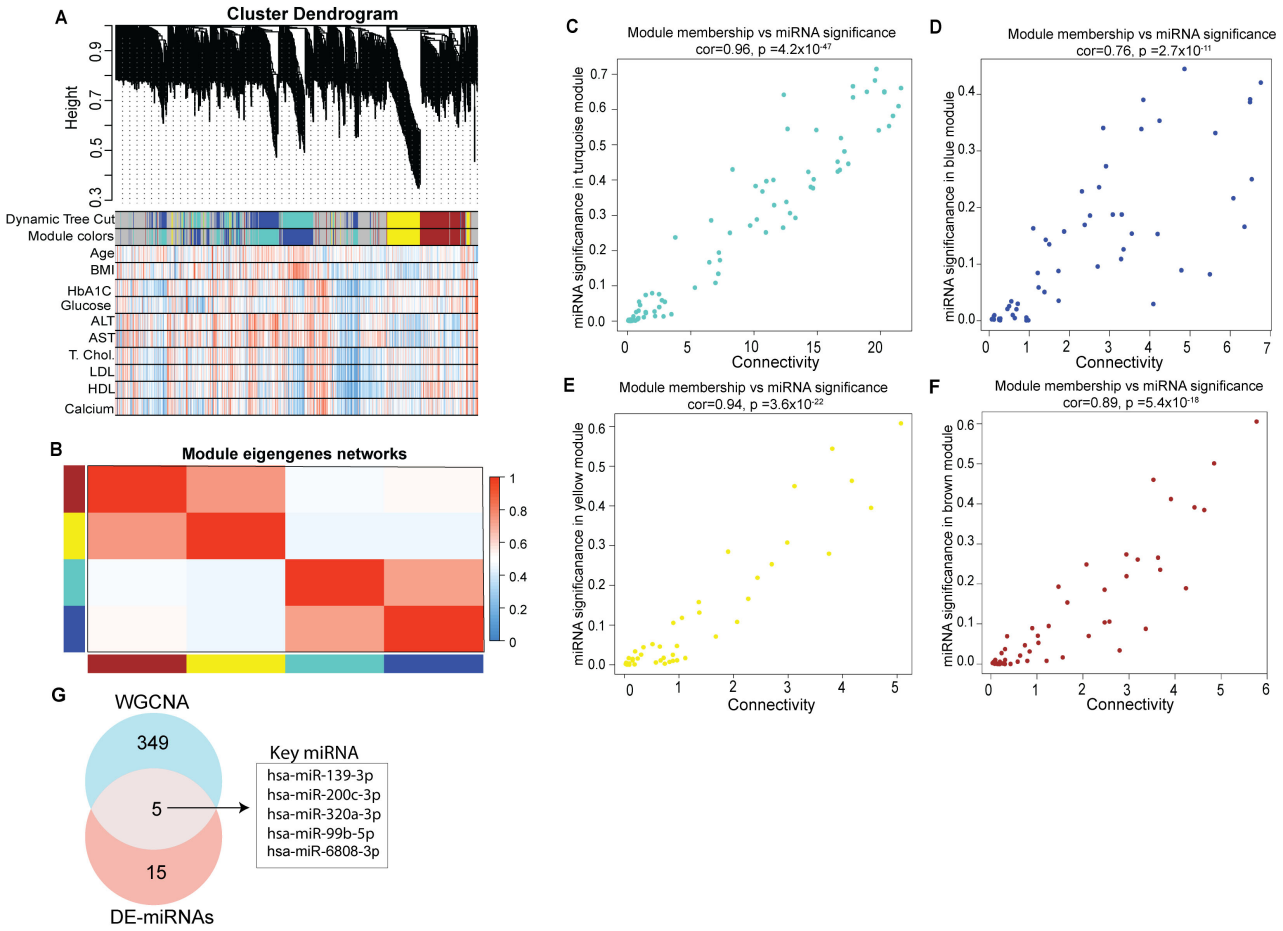
Identification of key modules by weighted gene co-expression network analysis. **(A)** Clustering dendrogram of miRNAs, with dissimilarity based on topological overlap, together with assigned module colors. Relationships of consensus module Eigen miRNAs (MEMs) and clinical traits status (age, BMI, HbA1C, Glucose, ALT, AST, Total cholesterol, LDL, HDL and calcium). Each row in the table corresponds to a module and each column to a clinical trait. The table is color-coded for correlation: red color indicates a positive while blue indicates a negative correlation. **(B)** Eigengene adjacency heatmap of different co-expression modules. **(C–F)** Correlation between module membership (MM) and connectivity of all miRNAs in each module. The scatter plot of eigengenes in tortoise, blue, yellow, and brown module, respectively. The figure shows the scatter plot of connectivity (x-axis) vs. MM (y-axis) in each module. **(G)** Venn diagram representing intersections among key module miRNAs and DE miRNAs.

We performed weighted gene co-expression network analysis to identify key modules and hub miRNAs involved in T1D (**Supplementary Table S5**). Correlation was used as a measure of miRNA expression on the data set consisting of 615 unique miRNAs detected across all the tested samples to identify significantly enriched modules (. The highly representative miRNA in each module was referred to as the module Eigen miRNA (MEM). **Figure 4** presents the co-expression module visualized as hierarchical *c*luster dendrograms and trait heatmap (**Figures 4A, B**). The clustering and dynamic tree cut algorithm resulted in five color-coded modules corresponding to grey, brown, blue, turquoise and yellow. The grey module was excluded from the analysis as it represents unassigned miRNAs. The blue module represents a total of 114, turquoise 76, yellow 80, and brown 84 distinct miRNAs; thus a total of 354 miRNAs were seen to form significant Eigen miRNA modules. Clinical traits (such as age, BMI, HbA1C, plasma glucose, serum alanine transaminase (ALT), aspartate transferase (AST), total cholesterol, LDL, HDL, and calcium) correlated with the miRNA expression in these five modules. For each miRNA in these four modules, the module significance (MS), module membership (MM), and intra-module connectivity (KME) were calculated to draw the scatterplots. Results indicated that MS was positively correlated with MM in all the four modules (**Figures 4C–F**) with correlation coefficients of 0.96, 0.94, 0.89, and 0.76 for the turquoise, yellow, brown, and blue module, respectively. Results from the analysis of DE miRNAs between T1D individuals and healthy individuals can be integrated with results from correlation network analysis to identify more precise targets (**Figure 4G**). A total of 5 miRNAs were seen common between the 18 DE miRNAs in T1D patients compared to healthy subjects and the 354 miRNAs forming the four significant Eigen miRNA modules.

These 5 miRNAs could be considered as playing a potential regulatory role in T1D (**Figure 4G**). These miRNAs were hsa-miR-200-3p, hsa-miR-139-3p, hsa-miR-320a-3p, hsa-miR-6808-3p and hsa-miR-99b-5p.

### 3.2 Mapping the key regulatory network in T1D

**Figure 5:**
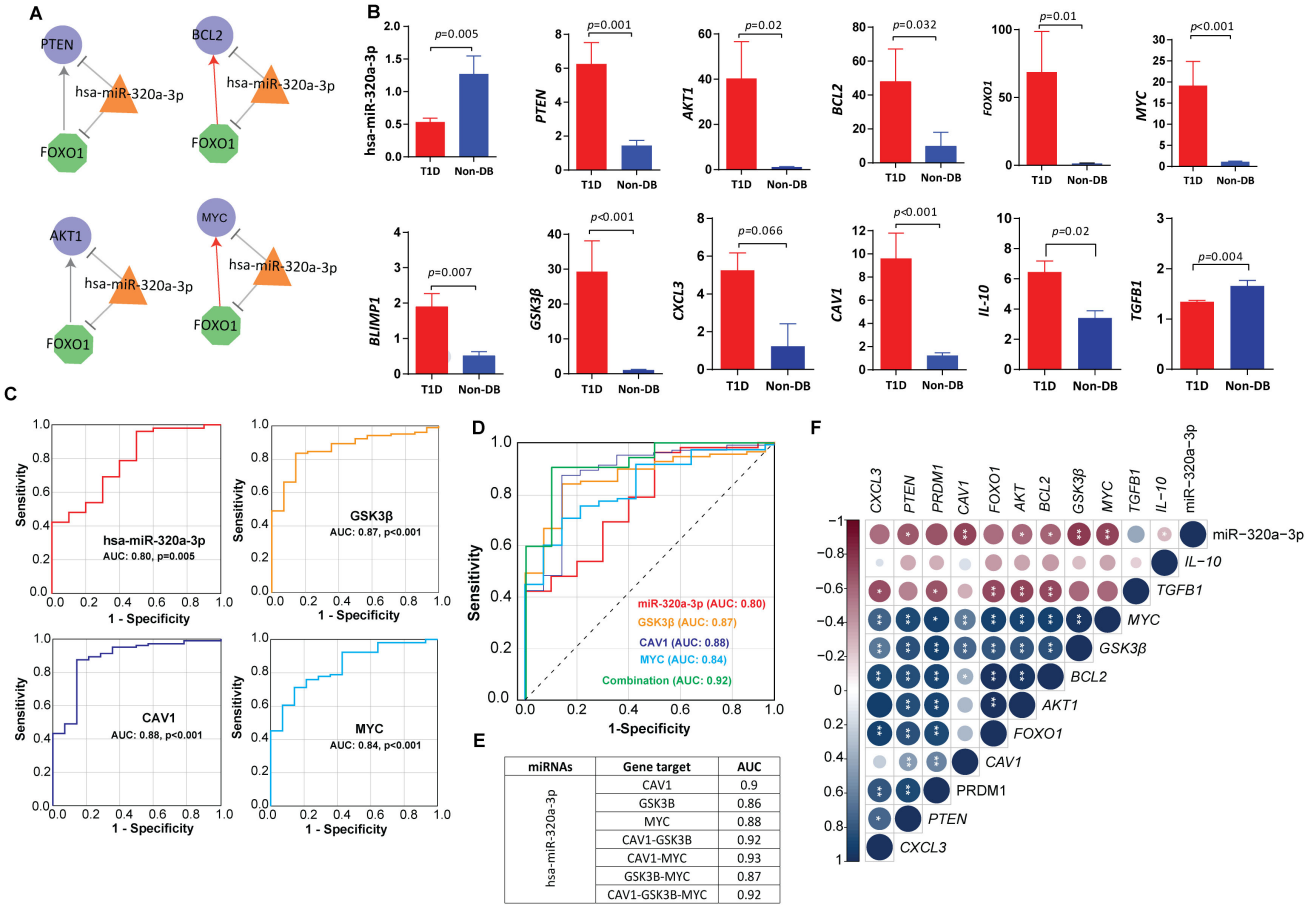
Circulating miRNA-mRNA biomarkers shortlisted by our study **(A)** miRNA feedforward loop (FFL) consisting of transcription factors-hsa-miR-320a-3p regulatory network. Nodes: The hexagonal shaped green nodes represent transcritpion factors (TFs), the triangle-shaped orange nodes represent miRNAs, and the circular-shaped blue nodes represent the targeted genes; Edges: sharp arrow means activation; T-shaped arrow represents repression. **(B)** Expression fold change of hsa-miR-320a-3p and other key targets in T1D versus healthy control, validated by quantitative real time PCR in sporadic T1D cases. The fold change (FC) was calculated using the 2 − ΔΔCT method, and differences in the expression levels between the two tested groups were detected using Mann-Whitney U-test. **(C)** The ROC curve analysis indicating the diagnostic potential of hsa-miR-320a-3p (AUC 0.83, *p-*value=0.005), *CAV1* (AUC: 0.88, *p-value<*0.001), *GSK3B* (AUC: 0.87, *p-value<*0.001) and *MYC* (AUC: 0.84, *p-value<*0.001). **(D, E)** Depicts the predictive power of hsa-miR-320a-3p in combination with *CAV1, GSK3B* and *MYC.* Using logistic regression, a linear combination of all four biomarkers, hsa-miR-320a-3p with *CAV1, GSK3B* and *MYC* led to an enhanced predictive accuracy of 0.92. **(F)** Depicts the correlations between hsa-miR-320a-3p and mRNA markers. Correlations between variables were calculated using Spearman’s rank correlation test and were considered statistically significant at *p*-value <0.05. Blue dots indicate positive correlation and red indicates negative correlation. * indicates p-value <0.05, ** indicates p-value <0.01.

We further investigated one of the shortlisted hub miRNAs, namely hsa-miR-320a-3p (MIMAT0000510), for its regulatory role by constructing the miRNA‐TF and the miRNA‐TF‐gene FFLs. The biological connectivity of 3-node motifs in TF, mRNA, and miRNA key regulatory networks are shown in **Figure 5A**. The highest-order network motif consisted of hsa-miR-320a-3p with *MYC* and *FOXO1* as the key transcription factors regulating the expression of miRNA target genes including *PTEN, BCL2*, and *AKT1.*

We measured the expression levels of the shortlisted hub miRNAs in an extended cohort of sporadic T1D children and ethnically-matched healthy children using targeted quantitative miRNA expression analysis. Expression of hsa-miR-320a-3p was observed to be significantly downregulated in children with T1D compared with non-diabetic controls (p-value=0.005) (**Figure 5B**). GO analysis of the hsa-miR-320a-3p hub miRNA shows enrichment of PI3K-AKT, MAPK and RAP1 signaling pathways. Results of the ROC analysis (**Figure 5C**) indicated the suitability of hsa-miR-320a-3p as a biomarker for T1D with an area under the curve (AUC) of 0.83, and asymptomatic *p-value=*0.005.

We further validated miRNA‐TF feedback loops and miRNA‐TF‐gene FFLs by targeted gene expression (**Figure 5B**). We observed significantly increased expression of key transcription factors namely *MYC (p-value<0.001)* and *FOXO1 (p-value=0.006)* in people with T1D compared with non-diabetic controls. In a similar manner, we observed a significant increase in the expression of hub genes such as *PTEN (p-value=0.02), AKT (p-value=*0.02), and *BCL2 (p-value=0.009)* in people with T1D compared with non-diabetic controls. Expression fold change of tested targets failed to show any significant correlation with clinical characteristics of study subjects such as age, sex, BMI, HbA1c, and autoantibody titers of IA-2 or GAD (*p*-value<0.05) at mRNA level (*p*-value>0.05).

We observed an inverse correlation between expression fold change of hsa-miR-320a-3p and shortlisted candidate genes such as *MYC (p-value=0.005)* and *BCL2 (p-value=0.034)* (**Supplementary Table S2**; **Figure 5F**). The hsa-miR-320a-3p also showed significant inverse correlation with insulin receptor-mediated signaling targets, such as *GSK3B (p-value=0.002)* and *CAV1 (p-value=0.003)*, and additionally with the anti-inflammatory marker IL-10 (*p-value=*0.03). We further observed a moderate to strong direct correlation between the expression of *MYC, FOXO1, PTEN, AKT* and *BCL2* and the expression of B-cell differentiation marker *BLIMP1 (p-value<0.001)* and the macrophage inflammatory marker *CXCL3 (p-value<0.001)* and additionally with the insulin signaling marker such as *GSK3B (p-value<0.001).* Consistently, the expression of *MYC* and *PTEN also* showed significant direct correlations with *CAV1 (p-value<0.003).* Expressions of *AKT, BCL2, and FOXO1* were inversely correlated with *TGFB1 (p-value<0.008)* (**Figure 5F**).

*CAV1, GSK3B*, and *MYC* are three potential markers that are differentially expressed at mRNA level, and their expression levels are significantly correlated with hsa-mir-320a-3p in T1D. ROC analysis (**Figure 5C**) indicated a predictive potential for *CAV1 (AUC: 0.88, p-value<0.001), GSK3B* (AUC:0.87, *p*-value<0.001), and *MYC* (AUC: 0.84, *p*-value<0.001) for T1D at the transcriptional level. A linear combination of hsa-miR-320a-3p with *CAV1*, *GSK3B* and *MYC* led to an enhanced predictive accuracy of 0.92 (**Figures 5D, E**).

We tested further whether the difference in the expression of hsa-miR-320a-3p stem from abnormal glucose and lipid metabolism by way of examining correlations between miRNA expression and the metabolic trait measurements in the study cohort. We observed no significant correlation between miRNA expression and any of the tested glucose or lipid parameters, with an exception of triglyceride (TGL) level. A moderately positive correlation was observed between hsa-miR-320a-3p expression and TGL (r=0.555, *p*-value=0.026). Given the fact that obesity and insulin secretion are interdependent, any correlation of hsa-miR-320a-3p seen with key glycemic and obesogenic targets may indicate its parellel role in diabetes and other metabolic complications.

Recent advances in long non-coding RNA (lncRNA) research indicate that lncRNA competes mRNA targets for miRNA binding sites, impacting gene expression. However, the role of lncRNA in T1D etiology is inadequate; as a preliminary attempt, we aimed to identify the key lncRNAs that interact with the shortlisted hsa-miR-320a-3p in human pancreatic tissue using DIANA-LncBaseV3.0 webtool. The hsa-miR-320a-3p was detected to target the expression profiles of *MEG3, NEAT1* and *AC015813.1* lncRNA genes, by way of adopting direct validation type and high confidence limit in human pancreatic tissue; this observation possibly indicate the significance of post-transcriptional regulatory events involving miRNA-lncRNA interaction in T1D, which needs to be further validated.

## 4 Discussion

The complexity and heterogeneity of T1D pose major challenges in identifying causative factors associated with the disease. A vast majority of T1D cases follow a polygenic model indicating a combined effect of multiple polymorphic genes and complex cellular mechanisms in the etiopathogenesis of the disease. In the present study, by way of examining both sib-pair and sporadic T1D cases from Kuwait, we highlight the key consensus miRNAs associated with T1D by primarily adopting differential miRNA analysis followed by a computational approach based on weighted gene co-expression network analysis. We evaluated key hub miRNA identified in the familial cohort for its potential to serve as a biomarker for T1D by validating it in an independent cohort of sporadic T1D cases by way of selecting the highest-order network motif consisting of hsa-miR-320a-3p, with *MYC* and *FOXO1* as the key transcription factors regulating the expression of key miRNA target genes such as *PTEN, BCL2*, and *AKT1.* We also provided additional evidence for the involvement of their key interacting partners such as *BLIMP1, GSK3B*, *CAV1, IL10* and *TGFB* in T1D pathogenesis.

The hsa-miR-320a-3p is one of the top prioritized miRNAs shortlisted by concatenated analyses of familial T1D cohort. Consistent with the observation from the familial cohort, the sporadic cases showed a significantly lower expression of hsa-miR-320a-3p in T1D patients compared with non-diabetic controls. Supportive evidence from literature also indicated a dysregulated expression of miR-320 in glucose and lipid metabolism (40, 41). Lower levels of hsa-miR-320 in blood have been associated with pre-diabetes and type 2 diabetes (T2D) in the Bruneck population in Italy (42). In contrast to these observations, a study by Karolina et al. (41) observed upregulation of hsa-miR-320 and a direct correlation between hsa-miR-320 and fasting blood glucose in the blood and exosomes of people with various metabolic conditions. Additionally, hsa-miR-320 is associated with cardiac dysfunction and lipotoxicity (40). Multiple studies have reported a dysregulated expression of hsa-miR-320a-3p in different types of carcinoma involving liver and pancreas (43, 44). Differences in the direction of mir-320 expression in various metabolic conditions hint towards the heterogeneity of the tested specimens and adopted technical methodologies.

**Figure 6:**
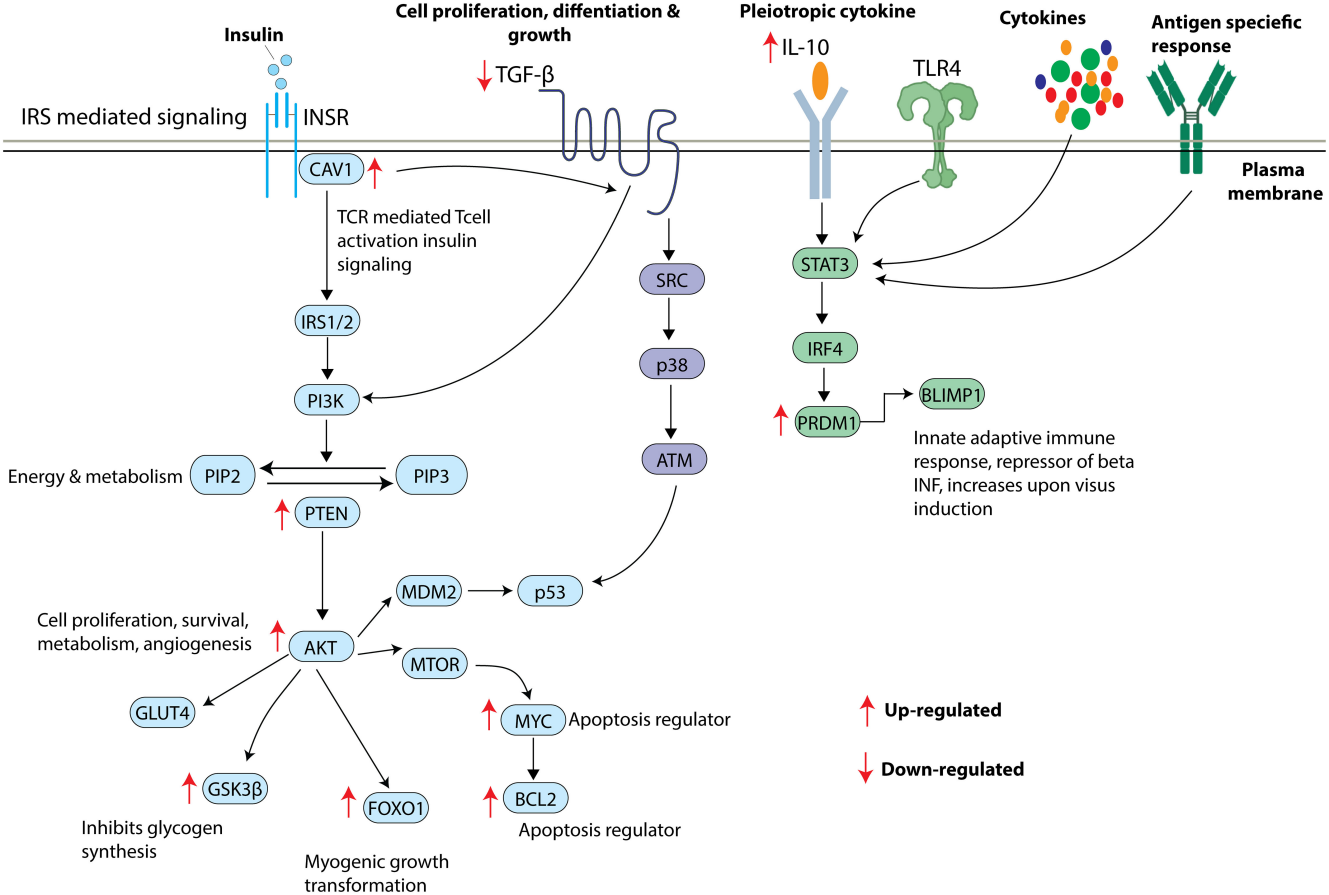
Overview of insulin signaling pathway and the shortlisted key mRNA markers. Insulin receptor stimulates *CAV1* and triggers a series of phosphorylation events activating IRS/PI3K/AKT pathway leading to increased *GLUT4* translocation, inhibition of glycogen synthesis, increased myogenic growth transformation, and increased apoptosis. *TGFB* also appears to significantly contribute to IRS/PI3K/AKT pathway influencing cell proliferation, differentiation, and growth. TL4R, cytokines and antigen specific responses lead to activation of *STAT3* resulting in the translation of *PRDM1* to BLIMP1 protein. Arrow indicates the direction of gene regulation.

To our knowledge, our study is the first to suggest the plausible association of hsa-miR-320a-3p with T1D etiology. Our results suggest a regulatory network comprising hsa-miR-320a-3p, along with key transcription factors and mRNA targets, to play a potential role in T1D etiology (**Figure 6**). We observed a significantly increased expression of two key transcription factors, namely *FOXO1* and *MYC* in our T1D cohort. Expression of hsa-miR-320a-3p was correlated inversely with that of *MYC*. The transcription factor *MYC* plays a detrimental role in intracellular glucose homeostasis and pancreatic beta cell function (45). Overexpression of *MYC* in cellular and animal models has led to increased beta cell proliferation, apoptosis and down-regulation of insulin gene leading to diabetes (46–49). *MYC* also tends to be a key regulator of major metabolic pathways, such as aerobic glycolysis, glutaminolysis, polyamine synthesis, and HIF-1α/mTOR (50). Similarly, the FOXO family of transcription factors plays a significant role in B lymphocyte maturation/function as part of adaptive immune response. Aberrant expression of FOXO family members has been widely associated with B cell malignancies (51). *FOXO1* plays a definitive role in the recombination activating genes (RAG) mediated immunoglobulin gene rearrangement (52). Though an increased expression of *FOXO1* was observed in our T1D cohort, it failed to show any significant correlation with that of hsa-miR-320a-3p.

Further, we observed a significantly increased expression of *PTEN, BCL2* and *AKT* in people with T1D compared with non-diabetic controls. The hsa-miR-320a-3p tended to be significantly associated with a decreased expression of target genes such as *PTEN, BCL2* and *AKT*. *PTEN* is a potent negative regulator of PI3K-AKT pathway, and its increased expression has been associated with key metabolic events characterizing diabetes (53, 54). Muscle targeted deletion of *PTEN* has been reported to protect mice from insulin resistance and diabetes caused by high-fat feeding (55). Deletion of *PTEN* in pancreatic beta cells leads to an increase in the beta cell mass, further implying the role of such a deletion in increased beta cell proliferation and diminished apoptosis (56). *BCL2* and *AKT* have been shown to have prominent roles in glucose and lipid metabolism (57–59). Pharmacological and genetic knockout of *BCL2* has been shown to profoundly improve glucose-dependent metabolic and ca2+ signaling in pancreatic cells (60).

GO analysis of the hsa-miR-320b hub miRNA also demonstrated enrichment of PI3K-AKT, MAPK and RAP1 signaling pathways. PI3K-AKT pathway has been considered as an emerging therapeutic target for T1D and beta cell dependent diseases (61, 62). The PI3K-AKT pathway is involved in diverse beta cell functions regulating the number of pancreatic islets, apoptosis, and cellular functions (63). It also tends to play a key role in the secretion of insulin by pancreatic beta cells (64). MAPK signaling pathway has also been considered as one of the key regulatory pathways involved in signal transductions related to viral replication and inflammatory cytokine synthesis, specifically with enterovirus infections (65) that are implicated in T1D pathogenesis (66). *RAP1* also plays a prominent role in glucose-stimulated islet cell insulin secretion, beta cell size and proliferation (67).

Our study also reported a deregulated expression of key interacting partners of hsa-miR-320a-3p namely *BLIMP1, GSK3B, CAV1, IL-10* and *TGFB1* in T1D at mRNA level (p-value<0.05). Higher levels of *BLIMP1* significantly correlated with the expression levels of *MYC, FOXO1, PTEN, AKT* and *BCL2* in T1D. *BLIMP1* is a candidate gene involved in key regulatory mechanisms involving T cell and B cell differentiation, immunoglobulin secretion and cytokine response (68–71). *GSK3B* is yet another candidate marker that correlates inversely with hsa-miR-320a-3p and directly with the target regulatory network highlighted in our study. *GSK3B* tends to dysregulate glucose homeostasis and is known for its potential role in promoting inflammation, endoplasmic reticulum stress, mitochondrial dysfunction, and apoptosis (72). Hence, we assume that the augmented expression of *GSK3B* may critically contribute to impaired glycemic control in people with T1D. Several lines of evidence indicate the role of *CAV1* in insulin secretion and insulin signaling (73) in diabetes and metabolic syndrome (73, 74). The hsa-miR-320a-3p tended to correlate inversely with *CAV1* in our study, further implying its significance in the pathogenesis of T1D. An upregulation of IL-10 may possibly be a counter-mechanism to combat hyper-inflammatory conditions (75). The reduction in the expression of *TGFB1* in T1D is significant; *TGFB1* has a prominent role in the development of pancreas and islet cell proliferation, differentiation, and apoptosis (76). Supportive evidence from literature indicates the protective effect of *TGFB1* on diabetes development. Overexpression of *TGFB1* under a rat insulin promoter reduces the risk of diabetes in T1D susceptible nonobese diabetic mice (77).

Additionally, our study highlights the interaction of hsa-miR-320a-3p with key lncRNAs targeting *MEG3, NEAT1* and *AC015813.1* genes in human pancreatic tissue. Interestingly, *MEG3* gene region was previously shown to be associated with susceptibility to T1D (78). In mouse model studies, a reduced expression of *MEG3* lncRNA in pancreatic beta cells tends to impact insulin synthesis and secretion (79). *MEG3* has also been shown to modulate key endothelial functions by interacting with other candidate markers such as *TGFB1* and *FOXO1* (80, 81). Increased circulating expression of *NEAT1* lncRNA has been reported in type 2 diabetic patients (82), while the role of *AC015813.1* lncRNA in diabetes is not known.

We highlight the possible role of hsa-miR-320a in T1D etiology, which has not been previously reported in the literature. Our findings based on the cohort from Kuwait are interesting, given the fact that the incidence of T1D is considerably increasing in the Arab region. A previous study on systemic literature review on T1D (83), have highlighted 11 consistently deregulated circulating miRNA markers (such as miR-21-5p, miR-24-3p, miR-100-5p, miR-146a-5p, miR-148a-3p, miR-150-5p, miR-181a-5p, miR-210-5p, miR-342-3p, miR-375 and miR-1275) associated with the disease. However, none of the shortlisted miRNA markers from our study, with the exception of hsa-miR-21-5p, overlaps with the above-mentioned markers implying the possible relevance of ethnic factors and associated clinical heterogeneity. One of the limitations of our study is that we used peripheral blood mononuclear cells which may reflect generalized systemic dysfunctions. Nevertheless, we assume that the transcriptional deregulations in peripheral blood are possibly in harmony with those in pancreas as supported by the increasing evidences for the involvement of the shortlisted targets in the pathophysiology of T1D. Although our sample size is relatively small (discovery cohort: 8 Kuwaiti-Arab families, with 18 T1D affected members and 18 unaffected members, characterized by no parent-to-child inheritance pattern; validation cohort: 110 people with T1D and 15 controls from which 52 sporadic T1D children and 10 ethnically-matched controls used for the validation of shortlisted miRNAs; and the entire validation cohort used for the validation of mRNA markers), there was sufficient power to reveal statistically significant novel results. A Power analysis of DE genes specifically in the control group showed an empirical power of >90%, presumably due to higher abundance of transcripts represented by these targets. It is noteworthy that these targets were shortlisted by way of adopting independent analysis strategies involving NGS-based differential expression analysis, module-based weighted gene co-expression network analysis in equal number of sib-pairs with and without T1D, and additionally by valdiation using targeted gene expression analysis. Our study warrants further in-depth validation in larger multi-ethnic age-matched cohorts, to reduce the confounding effect of age and ethnicty on the obtained results.

In conclusion, our study highlights the prospective role of hsa-miR-320a-3p in the pathophysiology of T1D by presenting known evidences for dysregulated expression of miRNA target-transcription factor network involving *PTEN, AKT1, BCL2, FOXO1* and *MYC.* The correlations of hsa-miR-320a-3p with additional interacting partners indicate its wide potential in insulin signaling and metabolic pathways characterizing the development of T1D. We highlight hsa-miR-320a-3p, *CAV1, GSK3B* and *MYC* as novel key biomarkers for T1D, and we further portray predictive transcriptional signatures of the key target mRNA-transcription factors associated with T1D. These observations lay the foundation for further in-depth research on catering to a better outcome and treatment of T1D.

## Data Availability

The datasets presented in this study can be found in online repositories. The names of the repository/repositories and accession number(s) can be found below: PRJNA1067840 (SRA).

## Supplementary material

Supplementary Figure 1 Determination of soft-thresholding power in the WGCNA. **(A)** Clustering dendrogram of samples based on their Euclidean distance, along with a heatmap of the clinical variables associated with each sample. **(B)** The plot shows the scale-free topology fit index (y-axis) for different soft-thresholding powers (β) (x-axis). **(C)** Analysis of the mean connectivity (degree, y-axis) for various soft-thresholding powers (x-axis). Clustering dendrogram of samples based on their Euclidean distance and heatmap of the clinical variables associated with each sample.
