# Supplementary figures and images for: Circulating hsa-miR-320a and its regulatory network in type 1 diabetes mellitus
Source: Front Immunol. 2024 Oct 11;15:1376416. doi: 10.3389/fimmu.2024.1376416 (PMC11502356; doi:10.3389/fimmu.2024.1376416)

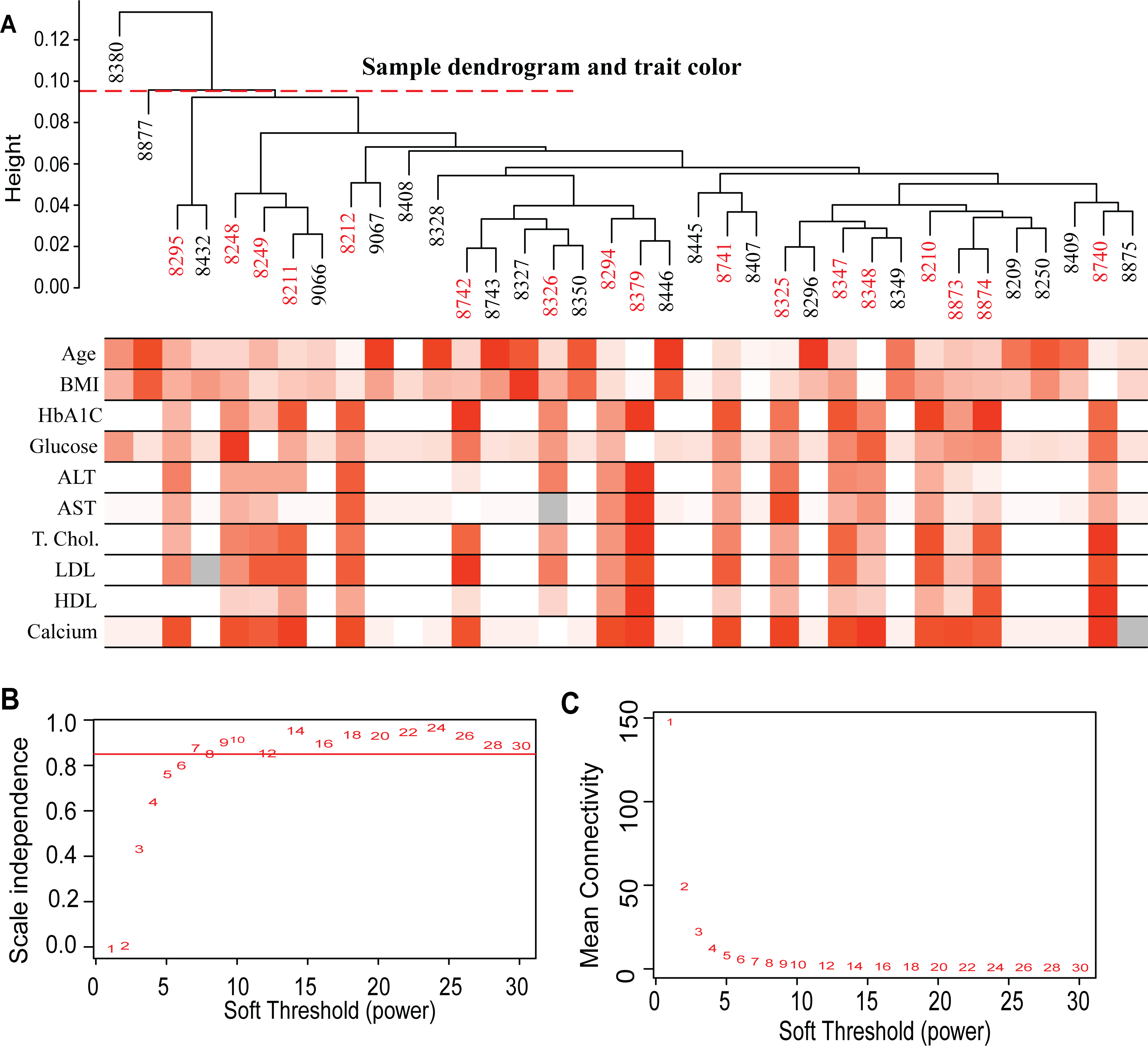

Supplement: Supplementary Figure 1 — Determination of soft-thresholding power in the WGCNA. (A) Clustering dendrogram of samples based on their Euclidean distance, along with a heatmap of the clinical variables associated with each sample. (B) The plot shows the scale-free topology fit index (y-axis) for different soft-thresholding powers (β) (x-axis). (C) Analysis of the mean connectivity (degree, y-axis) for various soft-thresholding powers (x-axis). Clustering dendrogram of samples based on their Euclidean distance and heatmap of the clinical variables associated with each sample. [file Image1.tif]
