# Supplementary material for: Circulating hsa-miR-320a and its regulatory network in type 1 diabetes mellitus
Source: Front Immunol. 2024 Oct 11;15:1376416. doi: 10.3389/fimmu.2024.1376416 (PMC11502356; doi:10.3389/fimmu.2024.1376416)
Supplement: Supplementary file 2 [file Table1.docx]

**Supplementary Table S1: Summary statistics of** **sequence reads of each sample tested in our study.**

| read set | S1 | S2 | S3 | S4 | S5 | S6 | S7 | S8 | S9 | S10 | S11 | S12 |
| --- | --- | --- | --- | --- | --- | --- | --- | --- | --- | --- | --- | --- |
| total_reads | 2,133,489 | 2,463,442 | 2,641,140 | 2,448,936 | 2,336,609 | 2,337,112 | 2,243,340 | 2,280,741 | 2,303,413 | 2,024,885 | 2,521,132 | 2,296,464 |
| no_adapter_reads | 216,044 | 379,545 | 196,929 | 328,882 | 373,460 | 340,025 | 306,042 | 288,134 | 286,833 | 457,016 | 372,597 | 307,582 |
| too_short_reads | 812,653 | 458,146 | 1,468,785 | 383,052 | 368,575 | 276,067 | 796,587 | 172,556 | 246,348 | 577,592 | 188,302 | 241,433 |
| UMI_defective_reads | 362,026 | 806,725 | 290,185 | 489,996 | 645,391 | 670,343 | 219,919 | 615,460 | 518,847 | 369,987 | 900,010 | 647,358 |
| miRNA_Reads | 404,425 | 491,810 | 354,214 | 857,069 | 630,437 | 649,041 | 568,122 | 765,980 | 760,580 | 391,239 | 609,964 | 627,950 |
| hairpin_Reads | 844 | 963 | 995 | 1,400 | 709 | 1,269 | 1,145 | 1,439 | 2,886 | 519 | 1,864 | 1,389 |
| piRNA_Reads | 21,568 | 24,186 | 15,619 | 33,121 | 28,355 | 29,050 | 22,992 | 30,088 | 31,992 | 16,995 | 28,438 | 28,504 |
| rRNA_Reads | 60,251 | 45,191 | 52,079 | 61,437 | 54,430 | 59,676 | 59,982 | 66,611 | 75,864 | 34,400 | 67,420 | 81,548 |
| tRNA_Reads | 21,565 | 14,286 | 17,222 | 23,735 | 16,836 | 15,140 | 14,012 | 22,577 | 33,950 | 20,522 | 15,088 | 50,401 |
| mRNA_Reads | 18,792 | 16,880 | 32,504 | 26,071 | 12,510 | 21,492 | 33,574 | 26,910 | 26,239 | 9,375 | 27,450 | 27,456 |
| otherRNA_Reads | 134,040 | 165,055 | 94,770 | 158,582 | 140,866 | 200,967 | 112,482 | 220,661 | 237,008 | 76,460 | 234,775 | 195,760 |
| notCharacterized_Mappable | 34,908 | 29,973 | 47,331 | 43,844 | 26,192 | 37,596 | 55,590 | 42,497 | 44,674 | 22,322 | 41,143 | 44,579 |
| notCharacterized_notMappable | 46,373 | 30,682 | 70,507 | 41,747 | 38,848 | 36,446 | 52,893 | 27,828 | 38,192 | 48,458 | 34,081 | 42,504 |
| read set | S13 | S14 | S15 | S16 | S17 | S18 | S19 | S20 | S21 | S22 | S23 | S24 |
| total_reads | 1,602,542 | 1,638,785 | 1,532,380 | 1,541,122 | 1,768,948 | 1,644,163 | 1,869,907 | 1,711,443 | 1,905,445 | 1,882,370 | 1,901,622 | 1,799,131 |
| no_adapter_reads | 279,798 | 184,926 | 208,677 | 190,609 | 245,008 | 270,941 | 233,037 | 261,954 | 163,252 | 219,971 | 281,237 | 220,644 |
| too_short_reads | 185,046 | 196,116 | 146,162 | 142,113 | 169,354 | 155,599 | 214,019 | 210,398 | 407,289 | 236,667 | 206,107 | 231,889 |
| UMI_defective_reads | 125,620 | 414,082 | 404,713 | 270,238 | 383,303 | 554,487 | 584,865 | 221,226 | 299,142 | 566,108 | 324,638 | 236,604 |
| miRNA_Reads | 709,545 | 460,914 | 499,677 | 630,743 | 613,879 | 402,990 | 426,464 | 548,745 | 591,106 | 450,979 | 661,169 | 730,556 |
| hairpin_Reads | 1,090 | 1,132 | 817 | 1,646 | 1,015 | 725 | 1,222 | 1,589 | 1,604 | 1,206 | 1,423 | 1,330 |
| piRNA_Reads | 26,880 | 26,481 | 25,889 | 28,261 | 29,970 | 22,207 | 23,256 | 29,920 | 31,252 | 26,626 | 32,495 | 30,975 |
| rRNA_Reads | 37,408 | 57,928 | 52,595 | 52,477 | 62,175 | 42,697 | 57,333 | 70,796 | 64,402 | 54,082 | 77,964 | 67,974 |
| tRNA_Reads | 40,292 | 34,850 | 23,877 | 24,841 | 26,841 | 15,142 | 55,471 | 52,277 | 56,972 | 52,191 | 81,842 | 58,002 |
| mRNA_Reads | 19,738 | 25,108 | 9,473 | 15,909 | 16,309 | 11,339 | 21,992 | 31,735 | 26,765 | 22,484 | 19,506 | 17,532 |
| otherRNA_Reads | 109,735 | 154,462 | 120,896 | 130,015 | 154,996 | 118,409 | 172,740 | 172,789 | 155,473 | 172,023 | 143,832 | 132,778 |
| notCharacterized_Mappable | 39,872 | 45,380 | 19,867 | 29,972 | 31,287 | 22,346 | 37,843 | 65,994 | 54,035 | 39,919 | 34,860 | 34,062 |
| notCharacterized_notMappable | 27,518 | 37,406 | 19,737 | 24,298 | 34,811 | 27,281 | 41,665 | 44,020 | 54,153 | 40,114 | 36,549 | 36,785 |
| read set | S25 | S26 | S27 | S28 | S29 | S30 | S31 | S32 | S33 | S34 | S35 | S36 |
| total_reads | 1,201,578 | 1,412,893 | 1,564,127 | 1,697,907 | 1,580,578 | 1,855,528 | 1,727,805 | 1,834,391 | 1,820,575 | 2,255,295 | 2,145,244 | 2,696,643 |
| no_adapter_reads | 214,733 | 264,049 | 211,708 | 181,211 | 222,625 | 349,504 | 304,396 | 306,144 | 379,037 | 249,166 | 241,301 | 353,265 |
| too_short_reads | 85,205 | 113,163 | 134,124 | 170,873 | 131,500 | 207,969 | 183,537 | 202,209 | 175,611 | 386,620 | 233,328 | 223,165 |
| UMI_defective_reads | 481,512 | 522,485 | 321,068 | 300,387 | 477,259 | 559,490 | 574,326 | 564,609 | 529,800 | 217,721 | 490,803 | 825,117 |
| miRNA_Reads | 252,698 | 271,626 | 615,483 | 683,375 | 399,633 | 475,906 | 304,596 | 474,334 | 475,067 | 981,595 | 627,004 | 767,208 |
| hairpin_Reads | 583 | 1,087 | 817 | 1,263 | 1,436 | 671 | 780 | 684 | 690 | 1,147 | 2,939 | 2,011 |
| piRNA_Reads | 16,706 | 21,531 | 35,316 | 35,392 | 28,035 | 34,844 | 24,129 | 43,223 | 29,883 | 46,504 | 38,009 | 54,344 |
| rRNA_Reads | 25,370 | 30,659 | 56,165 | 55,568 | 41,543 | 43,984 | 72,435 | 45,337 | 43,484 | 75,615 | 61,514 | 59,576 |
| tRNA_Reads | 12,431 | 12,902 | 16,086 | 48,537 | 12,269 | 16,910 | 18,580 | 30,931 | 14,978 | 22,208 | 67,111 | 18,439 |
| mRNA_Reads | 9,129 | 10,706 | 11,141 | 10,841 | 15,594 | 11,459 | 12,497 | 12,660 | 8,912 | 12,007 | 28,226 | 24,012 |
| otherRNA_Reads | 71,052 | 122,330 | 114,039 | 118,610 | 194,233 | 100,703 | 169,292 | 107,160 | 113,397 | 135,809 | 197,853 | 241,938 |
| notCharacterized_Mappable | 18,042 | 23,324 | 24,782 | 31,352 | 31,685 | 23,909 | 30,427 | 24,324 | 23,206 | 40,273 | 63,791 | 56,806 |
| notCharacterized_notMappable | 14,117 | 19,031 | 23,398 | 60,498 | 24,766 | 30,179 | 32,810 | 22,776 | 26,510 | 86,630 | 93,365 | 70,762 |

**Supplementary Table S2: Correlation matrix of mir-mRNA markers tested in our study.**

| Targets | Test* | **hsa-miR-320-3P** | ***CAV1*** | ***MYC*** | ***PRDM1*** | ***PTEN*** | ***GSK3B*** | ***CXCL3*** | ***AKT*** | ***BCL2*** | ***FOXO1*** | ***IL-10*** | ***TGFB1*** |
| --- | --- | --- | --- | --- | --- | --- | --- | --- | --- | --- | --- | --- | --- |
| **hsa-miR-320-3P** | Correlation Coefficient | 1.000 | -.368^**^ | -.350^**^ | -0.236 | -.251^*^ | -.385^**^ | -0.205 | -.255^*^ | -.274^*^ | -0.237 | -.276^*^ | 0.118 |
|  | *p*-value |  | **0.003** | **0.005** | 0.065 | **0.049** | **0.002** | 0.109 | **0.046** | **0.034** | 0.068 | **0.030** | 0.364 |
| ***CAV1*** | Correlation Coefficient | -.368^**^ | 1.000 | .500^**^ | .426^**^ | .270^**^ | .547^**^ | 0.131 | 0.169 | .204^*^ | 0.184 | 0.084 | -0.041 |
|  | *p*-value | **0.003** |  | **<0.001** | **<0.001** | **0.003** | **<0.001** | 0.158 | 0.069 | **0.038** | 0.055 | 0.367 | 0.659 |
| ***MYC*** | Correlation Coefficient | -.350^**^ | .500^**^ | 1.000 | .660^**^ | .624^**^ | .853^**^ | .494^**^ | .712^**^ | .712^**^ | .738^**^ | -0.133 | -0.117 |
|  | *p*-value | **0.005** | **<0.001** |  | **<0.001** | **<0.001** | **<0.001** | **<0.001** | **<0.001** | **<0.001** | **<0.001** | 0.153 | 0.209 |
| ***PRDM1*** | Correlation Coefficient | -0.236 | .426^**^ | .660^**^ | 1.000 | .580^**^ | .667^**^ | .579^**^ | .626^**^ | .615^**^ | .598^**^ | -0.068 | -.182^*^ |
|  | *p*-value | 0.065 | **<0.001** | **<0.001** |  | **<0.001** | **<0.001** | **<0.001** | **<0.001** | **<0.001** | **<0.001** | 0.463 | **0.050** |
| ***PTEN*** | Correlation Coefficient | -.251^*^ | .270^**^ | .624^**^ | .580^**^ | 1.000 | .551^**^ | .444^**^ | .576^**^ | .548^**^ | .527^**^ | -0.080 | -0.027 |
|  | *p*-value | **0.049** | **0.003** | **<0.001** | **<0.001** |  | **<0.001** | **<0.001** | **<0.001** | **<0.001** | **<0.001** | 0.394 | 0.774 |
| ***GSK3B*** | Correlation Coefficient | -.385^**^ | .547^**^ | .853^**^ | .667^**^ | .551^**^ | 1.000 | .347^**^ | .555^**^ | .606^**^ | .585^**^ | -0.057 | -0.158 |
|  | *p*-value | **0.002** | **<0.001** | **<0.001** | **<0.001** | **<0.001** |  | **<0.001** | **<0.001** | **<0.001** | **<0.001** | 0.543 | 0.089 |
| ***CXCL3*** | Correlation Coefficient | -0.205 | 0.131 | .494^**^ | .579^**^ | .444^**^ | .347^**^ | 1.000 | .699^**^ | .591^**^ | .692^**^ | 0.028 | -.200^*^ |
|  | *p*-value | 0.109 | 0.158 | **<0.001** | **<0.001** | **<0.001** | **<0.001** |  | **<0.001** | **<0.001** | **<0.001** | 0.764 | **0.031** |
| ***AKT*** | Correlation Coefficient | -.255^*^ | 0.169 | .712^**^ | .626^**^ | .576^**^ | .555^**^ | .699^**^ | 1.000 | .793^**^ | .814^**^ | -0.129 | -.309^**^ |
|  | *p*-value | **0.046** | 0.069 | **<0.001** | **<0.001** | **<0.001** | **<0.001** | **<0.001** |  | **<0.001** | **<0.001** | 0.166 | **0.001** |
| ***BCL2*** | Correlation Coefficient | -.274^*^ | .204^*^ | .712^**^ | .615^**^ | .548^**^ | .606^**^ | .591^**^ | .793^**^ | 1.000 | .871^**^ | -0.191 | -.285^**^ |
|  | *p*-value | **0.034** | **0.038** | **<0.001** | **<0.001** | **<0.001** | **<0.001** | **<0.001** | **<0.001** |  | **<0.001** | 0.053 | **0.004** |
| ***FOXO1*** | Correlation Coefficient | -0.237 | 0.184 | .738^**^ | .598^**^ | .527^**^ | .585^**^ | .692^**^ | .814^**^ | .871^**^ | 1.000 | -0.180 | -.290^**^ |
|  | *p*-value | 0.068 | 0.055 | **<0.001** | **<0.001** | **<0.001** | **<0.001** | **<0.001** | **<0.001** | **<0.001** |  | 0.060 | **0.002** |
| ***IL-10*** | Correlation Coefficient | -.276^*^ | 0.084 | -0.133 | -0.068 | -0.080 | -0.057 | 0.028 | -0.129 | -0.191 | -0.180 | 1.000 | -0.114 |
|  | *p*-value | **0.030** | 0.367 | 0.153 | 0.463 | 0.394 | 0.543 | 0.764 | 0.166 | 0.053 | 0.060 |  | 0.209 |
| ***TGFB1*** | Correlation Coefficient | 0.118 | -0.041 | -0.117 | -.182^*^ | -0.027 | -0.158 | -.200^*^ | -.309^**^ | -.285^**^ | -.290^**^ | -0.114 | 1.000 |
|  | *p*-value | 0.364 | 0.659 | 0.209 | **0.050** | 0.774 | 0.089 | **0.031** | **0.001** | **0.004** | **0.002** | 0.209 |  |

*Spearman correlation test
